# A hepatitis C-positive patient with new onset of nephrotic syndrome and systemic amyloidosis secondary to common variable immunodeficiency

**DOI:** 10.4103/0256-4947.67085

**Published:** 2010

**Authors:** Kultigin Turkmen, Melih Anil, Yalcin Solak, Huseyin Atalay, Hasan Esen, Halil Z. Tonbul

**Affiliations:** From the Selcuk University Meram School of Medicine, Nephrology Department, Konya, Turkey

## Abstract

Common variable immunodeficiency (CVID) is a heterogenous group of predominantly antibody-deficiency disorders that make up the greatest proportion of patients with symptomatic primary hypogammaglobulinemia. The rare coincidence of amyloidosis and hypogammaglobulinemia has been reported previously. Contrary to the usual insidious, slowly progressive disease following hepatitis C infection, a rapidly progressive cirrhotic form can develop in hypogammaglobulinemic patients. We report a HCV-positive patient with a new onset of nephrotic syndrome and systemic amyloidosis secondary to CVID. Blood analyses showed serum creatinine of 1.8 mg/dL and serum albumin of 3.1 gm/dL; 24-h urinary protein was 11 800 mg/day. Serum immunoglobulin levels were IgG 340 mg/dL, IgM 18 mg/dL, IgA 11 mg/dL. Duodenal biopsy revealed AA-type amyloidosis with potassium permanganate and Congo red staining. After a month of antiproteinuric therapy, the proteinuria was reduced to 3350 mg/day.

Hypogammaglobulinemia may be a primary genetic disease or a secondary disease that is due to other factors. For example, it may be the sequelae of certain infectious diseases, malignancy, various medications and systemic diseases.^1^ Common variable immunodeficiency (CVID) is the type of primary immunodeficiency that is most commonly encountered in clinical practice and is the second most common type of hypogammaglobulinemia. It is characterized by decreased levels of IgG, IgA and IgM secondary to impaired B cell differentiation. The patient may therefore have frequent respiratory tract infections, gastrointestinal and liver disease, granulomatous infiltration, unexplained hepatosplenomegaly, and an increased risk of malignancy and autoimmune diseases.^2^ CVID is a rare disorder that occurs at a rate of approximately 1 case per 100 000 births. The age at presentation of CVID has a bimodal distribution. Although the typical age of onset is 20 to 30 years, CVID may not become obvious until much later.^3^ Although amyloidosis is a rare complication of hypogammaglobulinemia, renal amyloidosis and systemic amyloidosis have been reported in patients with hypogammaglobulinemia, which has been associated with increased morbidity and mortality.^4^ Unlike the usual insidious, slowly progressive type of hepatitis C, a rapidly progressive cirrhotic form can develop in hypogammaglobulinemic patients. We report an HCV-positive patient with a new onset of nephrotic syndrome and systemic amyloidosis secondary to CVID.

## CASE

We admitted a 29-year-old male patient with complaints of dyspepsia, non-bloody mucous diarrhea and bilateral swelling of the ankles for 2 weeks. He had a 20-year history of recurrent upper and lower respiratory and gastrointestinal tract infections. He had been evaluated for these recurrent infections and hypogammaglobulinemia secondary to CVID had been diagnosed 9 years previously. At the time of diagnosis, the serum albumin level was in the normal range, but all types of serum immunoglobulins were below the normal values. On admission, his temperature was 38°C, and he had a dry tongue and decreased skin turgor and tonus. His blood pressure was 90/60 mm Hg and the heart rate was 84 beats/min, with a regular rhythm. Diffuse thyromegaly was evident on palpation. He had bilateral +++/+++ pretibial edema. Heart auscultation was unremarkable, and the lungs were clear. Hepatosplenomegaly was present. Blood and urine analyses showed serum creatinine: 1.8 mg/dL, serum albumin: 3.1 g/dL, AST: 35 IU/mL, ALT: 40 IU/mL, LDL-cholesterol: 170 mg/dL, triglycerides: 200 mg/dL, and 24-hour urinary protein: 11 800 mg/day. The hemogram showed white blood cell count: 6550/mL (neutrophil: 3700/mL and lymphocyte: 1850/mL), hemoglobin: 11 g/dL, and platelet count: 189 000/mL. HBs-Ag was negative, anti-HBs was positive (50 IU/L), anti-HCV was positive, HCV RNA: 1000 IU/mL (5200 copies/mL) (HCV RNA 3.0 assay, Versant Bayer); cutoff value for this assay is 615 IU/mL or 3200HCV RNA copies/mL. Serum immunoglobulin levels were as follows: IgG: 340 mg/dL (normal range, 750-1560), Ig-M: 18 mg/dL (normal range, 46-304), IgA: 11 mg/dL (normal range, 82-453). On the peripheral blood flow cytometry test, the proportion of cells expressing CD-19+ (20%), CD3+ (73%) and CD4+ (28%) were normal; however, CD8+ cells (47%) were increased. Anti-gliadin antibody and anti-endomysium IgA antibody were negative. The tuberculin skin test was negative (8 mm). Stool examination revealed *Giardia lamblia* cysts and trophozoites. Abdominal ultrasonography showed hepatosplenomegaly and bilaterally enlarged kidneys, without hydronephrosis. The patient had not had any symptoms, including the typical abdominal pain, which is the main symptom of familial Mediterranean fever (FMF). He also had no family history of FMF. In addition, mutations of the MEVF gene on exon 10 associated with FMF were negative.

**Figure 1:**
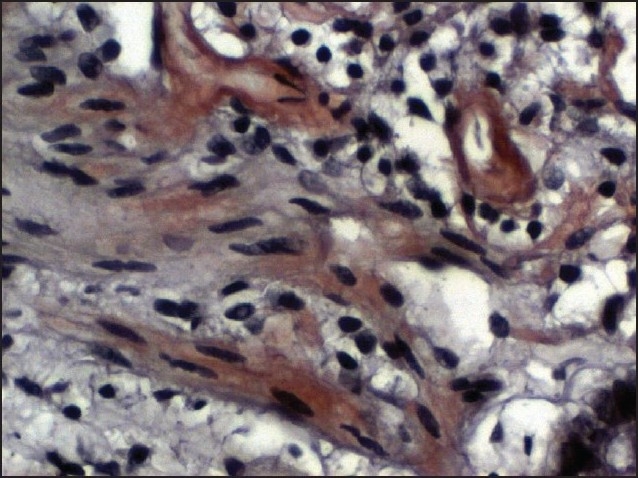
Duodenal biopsy specimen which showed AA Amyloidosis. Congo-red staining and 40× magnification.

To confirm that his complaints were indeed of new onset, we performed upper gastrointestinal endoscopy and duodenal biopsy. Gastroscopy was normal; duodenal biopsy showed AA type amyloidosis with potassium permanganate and Congo red staining (Figure 1) and duodenal lymphoid hyperplasia. Isotonic saline infusion (3000 mL/day), ciprofloxacin (200 mg bid) and metronidazole (500 mg tid) intravenously, and intravenous gammaglobulin 400 mg/kg/day were administered to treat the dehydration and active gastrointestinal infection. Both losartan (50 mg/day) and ramipril (2.5 mg/day) were also initiated to reduce the proteinuria. After a month of antiproteinuric therapy, the proteinuria was reduced to 3350 mg/day.

## DISCUSSION

CVID is a heterogenous group of disorders with both B-cell and T-cell dysfunctions. The main failure is in B-cell differentiation and immunoglobulin secretion. T-cell abnormalities have also been reported, including decreased lymphocyte proliferation in response to mitogens and antigens, deficiency of antigen-primed T cells, and reduced production and/or expression of cytokines, especially IL-2.^5^ Other changes usually seen in CVID include a decreased CD4/CD8 peripheral T-cell ratio secondary to increased CD8+ T-cells. Some studies have suggested that these CD8+ T-cells are functionally abnormal and suppress B- cell differentiation.^5^

CVID is associated with a wide spectrum of disorders including infection, chronic lung disease, autoimmune disease, gastrointestinal and liver disorders, granulomatous infiltration, splenomegaly, and an increased risk of malignancy.^5^ Infections, particularly pneumonia, sinusitis, and otitis media, are the most common features in hypogammaglobulinemia both before and after diagnosis, and affect 60% to 80% of patients; the organisms most commonly involved are *Streptococcus pneumoniae, Haemophilus influenzae, staphylococci,* and *Pseudomonas*. Diarrhea affects approximately 25% of patients, and is due to microorganisms such as *G lamblia, Rotavirus, Campylobacter, Enterovirus, Salmonella*, and *Shigella*.^6^

Because CVID is most commonly diagnosed in adulthood, many patients are likely to have previously received live vaccines for infectious diseases. Additional immunity is provided for patients treated with intravenous or subcutaneous immunoglobulin because circulating antibodies are present in these preparations. However, the measles--mumps--rubella and varicella vaccines are not recommended in patients receiving replacement immunoglobulin therapy because these vaccines may be inactivated by the presence of neutralizing antibodies. Inactivated vaccines can be given to patients with CVID, but these may not be effective because of the underlying antibody deficiency. Because influenza is unlikely to be represented in the replacement-immunoglobulin, the inactivated-subunit influenza vaccine is commonly recommended yearly as a prophylactic.^5^

Amyloidosis is a rare complication of hypogammaglobulinemia.^7^ Renal amyloidosis and systemic amyloidosis have been reported in patients with hypogammaglobulinemia and are associated with increased morbidity and mortality.^4^ None of the 96 X-linked agammaglobulinemia (XLA) patients reported by Lederman et al had amyloidosis, and only 2 of 44 XLA patients had renal amyloidosis in the study by Hermansky et al.^8^,^9^

In our case, the new onset of nephritic-range proteinuria, hypotension, diffuse thyromegaly, and hepatosplenomegaly were all consequences of systemic amyloidosis. To establish the diagnosis, a duodenal biopsy was performed and this revealed AA-type amyloidosis and duodenal lymphoid hyperplasia. Villous atrophy, duodenitis, and duodenal nodular hyperplasia can be observed in CVID patients, and there is an increased risk of malignancy in these patients as compared to the normal population. Unnecessary operative procedures should be avoided and, when necessary, there should be sufficient follow-up programs.^10^ Although we could not perform a kidney biopsy in our patient, a kidney biopsy is essential in an HCV-positive patients with CVID so as to exclude HCV-related nephritis. In our patient we excluded other reasons for the AA amyloidosis, such as rheumatoid arthritis, pulmonary tuberculosis, FMF, and other chronic diseases. Since massive proteinuria is associated with increased mortality and morbidity, vigorous antiproteinuric treatment modality is necessary, including angiotensin-converting enzyme inhibitors and/or angiotensin-receptor blockers. If proteinuria persists even after optimal therapy, pharmacological or interventional nephrectomy should be considered with non-steroidal anti-inflammatory drugs and renal artery embolization, respectively. In view of the rare coincidence of amyloidosis and hypogammaglobulinemia, it is possible that there may be additional factors such as (e.g., genetic or immunologic basis) for the development of amyloidosis.

Patients with CVID have low levels of antibody against HCV infection. Aiuti et al demonstrated in vitro that 8 of 18 HCV-infected patients were actively producing anti-HCV antibodies, despite their impaired antibody production. The high rate of HCV infection in hypogammaglobulinemic patients could be related to several nosocomial routes of transmission, including intravenous immune globulin administration.^11^

HCV is an indolent, often subclinical, disease that may lead to cirrhosis and hepatocellular carcinoma after many decades. However, in hypogammaglobulinemic patients with HCV, the disease progresses faster, leading to cirrhosis in approximately 10 years.^12^ Therefore, serological and clinical findings of HCV in CVID patients should be checked more closely than in patients without CVID.
